# Characteristics of Different Size Phytoplankton for Primary Production and Biochemical Compositions in the Western East/Japan Sea

**DOI:** 10.3389/fmicb.2020.560102

**Published:** 2020-12-21

**Authors:** Jae Joong Kang, Hyo Keun Jang, Jae-Hyun Lim, Dabin Lee, Jae Hyung Lee, Hyeonji Bae, Chang Hwa Lee, Chang-Keun Kang, Sang Heon Lee

**Affiliations:** ^1^Department of Oceanography, Pusan National University, Busan, South Korea; ^2^East Sea Fisheries Research Institute, National Institute of Fisheries Science, Gangneung, South Korea; ^3^South Sea Fisheries Research Institute, National Institute of Fisheries Science, Yeosu-si, South Korea; ^4^School of Earth Sciences and Environmental Engineering, Gwangju Institute of Science and Technology, Gwangju, South Korea

**Keywords:** primary production, biochemical compositions, small phytoplankton, large phytoplankton, calorific content, East/Japan Sea

## Abstract

The current phytoplankton community structure is expected to change, with small phytoplankton becoming dominant under ongoing warming conditions. To understand and evaluate the ecological roles of small phytoplankton in terms of food quantity and quality, the carbon uptake rates and intracellular biochemical compositions (i.e., carbohydrates, CHO; proteins, PRT; and lipids, LIP) of phytoplankton of different sizes were analyzed and compared in two different regions of the western East/Japan Sea (EJS): the Ulleung Basin (UB) and northwestern East/Japan Sea (NES). The average carbon uptake rate by the whole phytoplankton community in the UB (79.0 ± 12.2 mg C m^–2^ h^–1^) was approximately two times higher than that in the NES (40.7 ± 2.2 mg C m^–2^ h^–1^), although the average chlorophyll *a* (chl *a*) concentration was similar between the UB (31.0 ± 8.4 mg chl *a* m^–2^) and NES (28.4 ± 7.9 mg chl *a* m^–2^). The main reasons for the large difference in the carbon uptake rates are believed to be water temperature, which affects metabolic activity and growth rate, and the difference in euphotic depths. The contributions of small phytoplankton to the total carbon uptake rate were not significantly different between the regions studied. However, the rate of decrease in the total carbon uptake with increasing contributions from small phytoplankton was substantially higher in the UB than in the NES. This result suggests that compared to other regions in the EJS, the primary production in the UB could decrease rapidly under ongoing climate change. The calorific contents calculated based on biochemical compositions were similar between the small (1.01 ± 0.33 Kcal m^–3^) and large (1.14 ± 0.36 Kcal m^–3^) phytoplankton in the UB, whereas the biochemical contents were higher in the large phytoplankton (1.88 ± 0.54 Kcal m^–3^) than in the small phytoplankton (1.06 ± 0.18 Kcal m^–3^) in the NES. The calorific values per unit of chl *a* were higher for the large phytoplankton than for the small phytoplankton in both regions, which suggests that large phytoplankton could provide a more energy efficient food source to organisms in higher trophic levels in the western EJS.

## Introduction

Phytoplankton, as primary producers, play an important role in the food web as well as the biogeochemical cycling of aquatic ecosystems. Primary production by the phytoplankton community is an important factor in controlling the quantity of food sources for higher trophic level organisms and subsequently could affect the recruitment, biomass, and production of fishery resources (Whyte, 1987; Kleppel and Burkart, 1995; Kang et al., 2017). Intracellular biochemical compositions (i.e., carbohydrates, CHO; proteins, PRT; and lipids, LIP) of phytoplankton could provide helpful information related to their physiological status and the nutritional value of food available to grazers (Lee et al., 2020). According to previous studies, the different biochemical compositions of phytoplankton are closely connected with the nutritional status and survival strategies of zooplankton communities (Scott, 1980; Sterner et al., 1993; Hessen et al., 1997; Lindqvist and Lignell, 1997; Yun et al., 2015; Jo et al., 2017). The quantity and quality of food provided by phytoplankton can be largely affected by various environmental conditions, such as light conditions, major nutrient availability, and phytoplankton species composition (Morris et al., 1974; Mortensen et al., 1988; Kilham et al., 1997; Lee et al., 2017a). In particular, phytoplankton size structure is one of the major factors controlling the efficiency of the transfer of energy fixed by photosynthesis toward upper trophic levels or into the ocean’s interior (Legendre and Rassoulzadegan, 1996; Falkowski and Oliver, 2007; Finkel et al., 2010; Marañón et al., 2012). Marine ecosystems dominated by small phytoplankton have low carbon export rates due to slow sinking rates and intense microbial decomposition of organic matter, whereas high downward export fluxes and efficient transfer of food material (FM) through short food chains appear in systems dominated by large phytoplankton (Marañón et al., 2012). Several studies have reported that recent climate change could lead to an increase in the contribution of small phytoplankton to the total phytoplankton biomass; thus, determining the ecological role of small phytoplankton as primary producers providing basic food sources in marine ecosystems is important under ongoing warming conditions (Agawin et al., 2000; Morán et al., 2010; Hilligsøe et al., 2011; Mousing et al., 2014).

The East/Japan Sea (EJS), located in the northwestern Pacific Ocean, is one of the highly productive oceanic regions and is regarded as a “miniature ocean” due to its dynamic environmental conditions (i.e., upwelling, eddies, and fronts) (Kwak et al., 2014; Lee et al., 2017b). The EJS includes three deep (>2000 m) basins: the Ulleung Basin, Yamato Basin, and Japan Basin. Among these basins, the most productive region is the Ulleung Basin (UB), which is located in the southwestern part of the EJS (Kwak et al., 2014; Lee et al., 2017b). Recently, the EJS, including the UB, has experienced notable changes in its physicochemical properties, such as drastic increases in sea surface temperature and rapid ocean acidification (Kim et al., 2001; Kang et al., 2003). These changes could accompany variations in biological characteristics, especially in phytoplankton communities and, subsequently, upper trophic levels (Chiba et al., 2012; Kang et al., 2017; Lee et al., 2017b). Indeed, remarkable changes in the duration and intensity of the phytoplankton spring bloom in the EJS (Lee et al., 2014) and a significant decline in the annual primary production in the UB (Joo et al., 2014) were reported by previous studies. In addition, Lee et al. (2017b) found decreasing trends in primary productivity with increasing contributions from small phytoplankton to the total community in the northern EJS. However, few studies have focused on the role of small phytoplankton as primary producers and basic food sources in the EJS, especially in the UB, which is considered a biological hotspot in the EJS.

In this research, differences in primary production and biochemical compositions by phytoplankton size were analyzed at two regions in the western EJS (i.e., the UB and northwestern East/Japan Sea – NES) during the spring bloom season. The primary objective in this study was to evaluate the effect of contributions from small phytoplankton on the total primary production in the western EJS. The other objective was to compare the difference in the physiological status and energy efficiency of the small and large phytoplankton in the western EJS.

## Materials and Methods

### Sample Collection and Environmental Data

**FIGURE 1:**
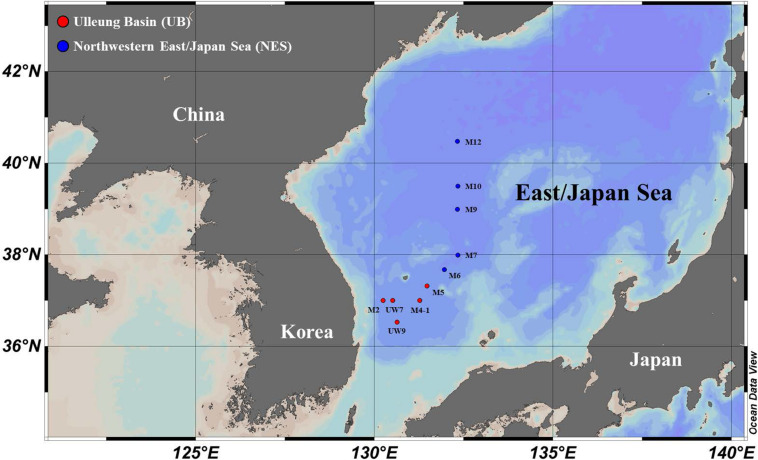
Locations of sampling stations for primary productivity and biochemical composition measurements in the western EJS in 2016.

Sampling of the carbon uptake rates of phytoplankton in the western EJS was carried out at 10 stations (UB: five stations and NES: five stations) selected from a total of 34 stations sampled during a joint Korean-Russian cruise conducted from 05 to 15 April 2016 (Figure 1 and Table 1). Temperature, salinity and density data were obtained using a CTD tool (conductivity, temperature, and depth tool; SBE 911 plus, Seabird Electronics Inc., Bellevue, WA, United States). The mixed layer depth (MLD) at each station was defined as the depth with a difference of 0.125 σ_t_ from the surface value (Gardner et al., 1995; Kwak et al., 2014). Water samples were collected to assess nutrient levels (nitrate, NO_3_; ammonium, NH_4_; phosphate, PO_4_; and silicate, SiO_4_) from three light depths (100, 30, and 1% penetration of surface photosynthetically active radiation, PAR) using Niskin bottles (12 L) attached to a CTD/rosette sampler at all the stations at which productivity was sampled. After the seawater samples were filtered through Whatman GF/F filters, each sample was immediately transferred into high-density polyethylene bottles (50 mL) and kept frozen at –80°C until analysis. The samples were returned to the home laboratory at Pusan National University, South Korea, and then nutrient concentrations were determined using an automated nutrient analyzer (Auto analyzer, Quaatro, Germany).

**TABLE 1:** Environmental variables, euphotic depth-integral chl *a* concentration and carbon uptake rates in the western EJS during the 2016 cruise.

											**Integrated Chl *a* (mg Chl *a* m^–2^)**		**Integrated carbon uptake rate (mg C m^–2^ h^–1^)**	
												
**Region**	**Station**	**Date (2016)**	**T_eu_ (°C)**	**S_eu_ (psu)**	**Z_eu_ (m)**	**Z_m_ (m)**	**NO_3_ (μM)**	**NH_4_ (μM)**	**SiO_2_ (μM)**	**PO_4_ (μM)**	**Small**	**Large**	**Small**	**Large**
Ulleung Basin (UB)	M2	07-April	12.9	34.4	51	49	1.47	0.62	1.55	0.22	26.7	18.4	34.9	36.1
	M4-1	08-April	13.8	34.5	33	28	1.10	0.60	2.01	0.24	16.3	10.7	36.4	43.9
	M5	09-April	14.5	34.5	35	56	0.74	0.45	1.26	0.12	10.5	12.7	39.3	59.3
	UW7	13-April	12.3	34.2	27	75	2.10	0.48	3.57	0.28	14.6	17.0	34.2	43.9
	UW9	14-April	13.4	34.4	27	88	1.34	0.53	3.18	0.15	19.0	9.4	32.2	34.8
North western EJS (NES)	M6	12-April	10.6	34.2	22	27	0.86	0.55	3.26	0.07	12.6	7.7	15.0	26.5
	M7	12-April	10.2	34.1	22	21	0.63	0.63	3.20	0.02	12.6	10.2	15.1	28.4
	M9	11-April	7.4	34.0	19	32	0.84	0.63	4.81	0.09	13.4	14.7	15.2	26.1
	M10	11-April	6.3	34.0	22	33	1.58	0.53	5.70	0.18	14.0	16.2	20.2	17.6
	M12	10-April	4.9	33.9	24	37	2.05	0.56	5.07	0.20	12.9	27.7	14.2	25.0

*T_*eu*_, water temperature averaged over the depth of the euphotic zone (Z_*eu*_); S_*eu*_, salinity averaged over Z_*eu*_; Z_*m*_, mixed layer depth.*

### Small and Large Size-Fractionated Chlorophyll *a* Measurement

Seawater samples for small (0.7–2.0 μm) and large (>2.0 μm) size-fractionated chlorophyll *a* (chl *a*) molecules were obtained from the three depths at which light was measured (i.e., 100, 30, and 1% PAR penetration). To estimate the size composition of the phytoplankton assemblages, the seawater samples were passed sequentially through a 2 μm (large chl *a*) Nuclepore membrane filter (47 mm) and then a 0.7 μm (small chl *a*) Whatman GF/F paper (47 mm). The filters were frozen immediately for further analysis in the laboratory. After extraction in 90% acetone, the concentrations of the size-fractionated chl *a* were determined with a previously calibrated fluorometer (Turner Designs model 10-AU) based on methods described by Kim et al. (2015).

### Measurement of the Carbon Uptake Rate of Phytoplankton

The carbon uptake rates of small (0.7–2.0 μm) and large (>2.0 μm) phytoplankton in the western EJS were measured with a ^13^C stable isotope technique. Seawater samples for carbon uptake rate measurement were obtained from six light depths (100, 50, 30, 12, 5, and 1%) at the selected productivity stations where incubation was available on deck under natural light conditions. A water sample from each of the different light depths was transferred into a polycarbonate incubation bottle (1 L) with a screen filter that created conditions corresponding to each light depth. A labeled carbon (NaH^13^CO_3_) solution, which corresponded to approximately 10% of the ambient concentration, was injected into all the incubation bottles to determine the carbon uptake rates of the phytoplankton (Dugdale and Goering, 1967; Hama et al., 1983; Lee et al., 2007, 2017b). The bottles were cultured in an acrylic incubator cooled by circulating surface seawater on deck for 4–5 h. To estimate the carbon uptake rate of the large phytoplankton, which was calculated as the difference in the carbon uptake rates of the total and small phytoplankton, the seawater samples used to assess the total phytoplankton carbon uptake rate from each incubation bottle were filtered through 25 mm GF/F filters after incubation. To assess the carbon uptake rate of the small phytoplankton, the seawater samples were first passed through a 2 μm Nuclepore filter (47 mm) to remove the large cells, and then the filtrate was passed through a 25 mm GF/F filter. The filters were immediately stored in a deep freezer for later mass spectrometer analysis. At the laboratory, acid fuming was applied overnight to all the samples for carbonate removal. The carbon stable isotope (^13^C) of the treated samples was measured using a Finnigan Delta + XL mass spectrometer at the stable isotope laboratory of the University of Alaska Fairbanks, United States. The carbon uptake rate was calculated following methods described by Hama et al. (1983).

### Biochemical Composition Measurements

To determine the biochemical compositions (carbohydrates, CHO; proteins, PRT; and lipids, LIP) of the phytoplankton, seawater samples were collected from three light depths (100, 30, and 1%) at seven stations selected from the 10 productivity stations (UB: three stations and NES: four stations). Each sample for the analysis of the total phytoplankton biochemical composition was filtered through 0.7 μm Whatman GF/F filters (47 mm). To evaluate the biochemical compositions of the small phytoplankton, additional water samples were passed sequentially through 2 μm Nucleopore membrane filters (47 mm) and 0.7 μm Whatman GF/F filters (47 mm). The biochemical compositions of the large phytoplankton were estimated as the difference in the compositions between the total and small phytoplankton. The filters were frozen immediately and preserved for further analysis at the laboratory. Each biochemical compound (CHO, PRT, and LIP) was analyzed at the laboratory based on the methods of Lowry et al. (1951); Dubois et al. (1956), and Bligh and Dyer (1959), respectively. The detailed methods used for analyzing each biochemical compound are described in Bhavya et al. (2019). FM represented the sum of the three biochemical components (CHO, PRT, and LIP), and calorific contents were calculated following Winberg (1971).

## Results

### Environmental Conditions

**FIGURE 2:**
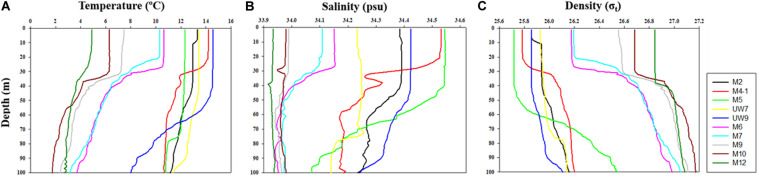
Vertical structures of **(A)** temperature, **(B)** salinity, and **(C)** density at all the experimental stations in the western EJS in 2016.

The hydrographic conditions were different between the UB and NES during the spring season in 2016. The MLD (derived from the density difference) at the UB stations had a relatively wide range, from 28 to 88 m (mean ± *SD* = 59.2 m ± 23.3 m), whereas the MLD at the NES stations ranged from 21 to 37 m, with a mean of 30.0 m (*SD* = ±6.2 m) (Figure 2 and Table 1). The MLDs at all the stations in the UB [except for station M4-1 (28 m)] were deeper (*t*-test, *p* < 0.05) than the those in the NES (Figure 2 and Table 1). The euphotic depths (i.e., the depth receiving 1% of the surface PAR) were also relatively deeper (*t*-test, *p* < 0.05) in the UB stations (range = 27–51 m; mean ± *SD* = 34.6 ± 9.8 m) than in the NES stations (range = 19–24 m; mean ± *SD* = 21.8 ± 1.8 m) (Table 1). Overall, the MLD was similar to the euphotic depth or deeper than the euphotic depth in both regions (Table 1), which indicates that the euphotic water columns were well mixed during our observation period (Figure 2). The water temperatures averaged over the depths of the euphotic zone in the UB ranged from 12.3 to 14.5°C (13.6 ± 0.9°C), whereas the average temperature within the euphotic zone in the NES ranged from 4.9 to 10.6°C (7.9 ± 2.5°C) (Table 1). The salinity ranged from 34.2 to 34.5 psu in the UB (34.4 ± 0.1 psu) and 33.9 to 34.2 psu in the NES (34.0 ± 0.1 psu) (Table 1). Temperature and salinity were higher (*t*-test, *p* < 0.01) in the UB than in the NES during the study period (Table 1).

**FIGURE 3:**
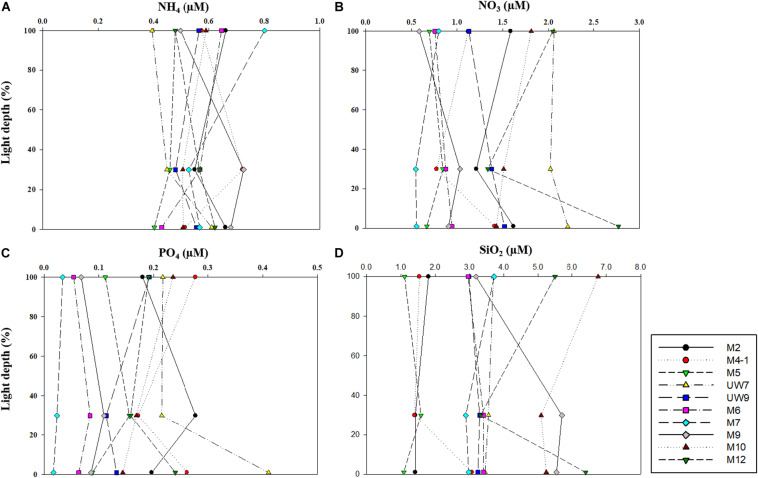
Concentrations of **(A)** ammonium (NH_4_), **(B)** nitrate (NO_3_), **(C)** phosphate (PO_4_), and **(D)** silicate (SiO_4_) within euphotic depth (100, 30, and 1% light depth) in 2016.

No noticeable differences in any of the major nutrient (NH_4_, NO_3_, PO_4_, and SiO_2_) concentrations were found at the different light depths (100, 30, and 1%) (one-way ANOVA, *p* > 0.05) in either region (Figure 3) since the water column within the euphotic zone was well mixed. The mean concentrations of all the major nutrients (NH_4_, NO_3_, and PO_4_) except for SiO_2_ averaged over the euphotic zone were not significantly different (*t*-test, *p* > 0.05) between the UB (NH_4_ = 0.54 ± 0.08 μM; NO_3_ = 1.35 ± 0.50 μM; PO_4_ = 0.20 ± 0.07 μM) and the NES (NH_4_ = 0.58 ± 0.05 μM; NO_3_ = 1.19 ± 0.60 μM; PO_4_ = 0.11 ± 0.07 μM) (Table 1). In contrast, the averaged SiO_2_ concentration was lower (*t*-test, *p* < 0.05) in the UB (mean ± *SD* = 2.31 ± 1.01 μM) than in the NES (mean ± *SD* = 4.41 ± 1.37 μM) (Table 1).

### Spatial Distribution of Phytoplankton Chl *a* Concentration in the UB and NES

**FIGURE 4:**
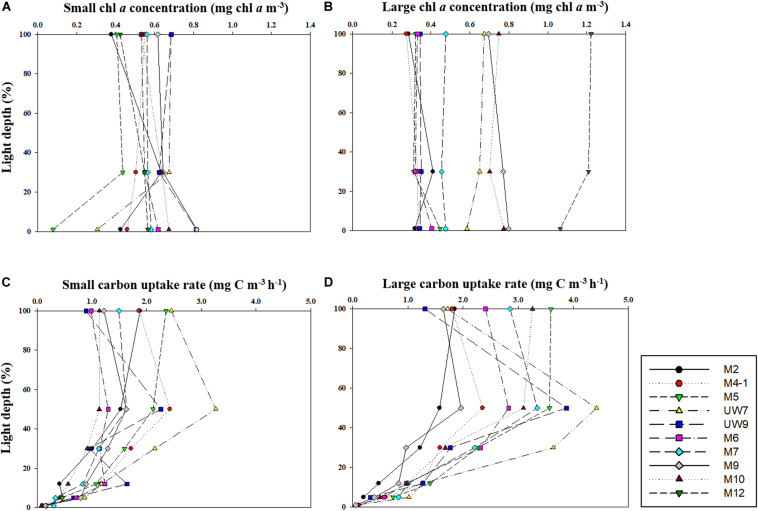
Vertical distributions of chl *a* concentration for **(A)** small and **(B)** large size phytoplankton and carbon uptake rates for **(C)** small and **(D)** large size phytoplankton at different light depths in 2016.

**FIGURE 5:**
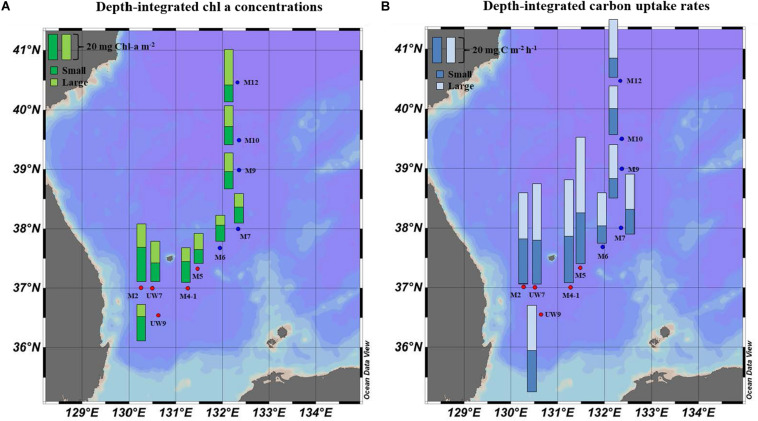
Spatial distributions of **(A)** chl *a* concentration and **(B)** hourly carbon uptake rates for small and large size phytoplankton integrated within the euphotic depths from 100 to 1% light depth at the productivity stations in 2016.

No distinct vertical differences in the concentrations of the total (sum of the small and large chl *a* concentrations), small or large chl *a* molecules were found within the euphotic depth at each station (one-way ANOVA, *p* > 0.05; Figures 4A,B) in this study. The total chl *a* concentration integrated from the surface to a depth with 1% light penetration in the UB ranged from 23.2 to 45.1 mg chl *a* m^–2^, with an average of 31.0 mg chl *a* m^–2^ (*SD* = ± 8.4 mg chl *a* m^–2^), and that in the NES ranged from 20.3 to 40.7 mg chl *a* m^–2^, with an average of 28.4 mg chl *a* m^–2^ (*SD* = ± 7.9 mg chl *a* m^–2^) (Figure 5A and Table 1). In the UB region, the average values of the concentrations of chl *a* from small and large phytoplankton integrated over depths with light penetration ranging from 100 to 1% were 17.4 ± 6.0 mg chl *a* m^–2^ (range: 10.5–26.7 mg chl *a* m^–2^) and 13.6 ± 3.9 mg chl *a* m^–2^ (range: 9.4–18.4 mg chl *a* m^–2^), which contributed 55.6 ± 9.5% and 44.4 ± 9.5% to the total chl *a* concentration, respectively (Figure 5A and Table 1). In contrast, the average chl *a* concentrations of the small and large phytoplankton over the same depth in the NES were 13.1 ± 0.6 mg chl *a* m^–2^ (range: 12.6–14.0 mg chl *a* m^–2^) and 15.3 ± 7.7 mg chl *a* m^–2^ (range: 7.7–27.7 mg chl *a* m^–2^), which contributed 48.6 ± 11.3% and 51.4 ± 11.3% to the total chl *a* concentration, respectively (Figure 5A and Table 1).

### Carbon Uptake Rates of Phytoplankton in the UB and NES

**FIGURE 6:**
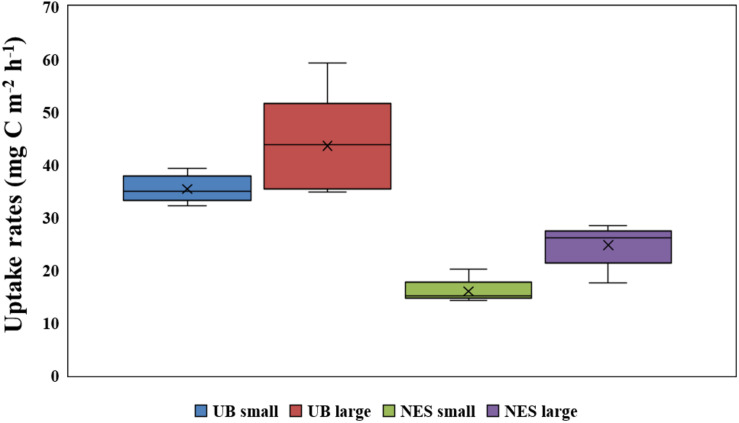
Comparison of depth-integral carbon uptake rates for small and large size phytoplankton in the UB and NES.

The hourly carbon uptake rates of the total, small and large phytoplankton communities within the euphotic water column differed at each light depth in the UB and the NES (Figures 4C,D). In general, the maximum hourly carbon uptake rates of the phytoplankton were observed in the surface layer (within a water depth of 10 m), which corresponded to a light depth of 50% (Figures 4C,D). The hourly carbon uptake rates of the total phytoplankton community integrated from the surface to the 1% light depth were approximately two times higher in the UB than in the NES during the observation period (Table 1). The range of the total carbon uptake rates from the surface to the 1% light depth was from 67.0 to 98.7 mg C m^–2^ h^–1^, with a mean of 79.0 mg C m^–2^ h^–1^ (*SD* = ± 12.2 mg C m^–2^ h^–1^) in the UB, whereas the range of the total hourly carbon uptake rates over the same depth in the NES was from 37.7 to 43.6 mg C m^–2^ h^–1^, with a mean of 40.7 mg C m^–2^ h^–1^ (*SD* = ± 2.2 mg C m^–2^ h^–1^) during the study period (Figure 5B and Table 1). The hourly carbon uptake rates of the small and large phytoplankton communities integrated from the surface to the 1% light depth in the UB ranged from 32.2 to 39.3 mg C m^–2^ h^–1^ (35.4 ± 2.7 mg C m^–2^ h^–1^) and from 34.8 to 59.3 mg C m^–2^ h^–1^ (43.6 ± 9.8 mg C m^–2^ h^–1^), respectively (Figure 6 and Table 1). In contrast, the integrated carbon uptake rates by the small and large phytoplankton communities in the NES ranged from 14.2 to 20.2 (15.9 ± 2.4 mg C m^–2^ h^–1^) and from 17.6 to 28.4 (24.7 ± 4.2 mg C m^–2^ h^–1^), respectively (Figure 6 and Table 1). The average carbon uptake rates (*t*-test, *p* < 0.05) of the small and large phytoplankton communities were higher in the UB than in the NES during our observation period (Figure 6 and Table 1). The contributions of small phytoplankton in the UB to the total carbon uptake rate ranged from 39.9 to 49.2%, with an average of 45.2% (*SD* = ± 3.7%), whereas those of large phytoplankton in the UB ranged from 50.8 to 60.1%, with an average of 54.8% (*SD* = ± 3.7%). In the NES, the small phytoplankton contributed 39.5 ± 7.8% (range: 34.8–53.4%) to the total phytoplankton carbon uptake rate, while the large phytoplankton contributed 60.5 ± 7.8% (range: 46.6–65.2%) (Figure 5B and Table 1).

**FIGURE 7:**
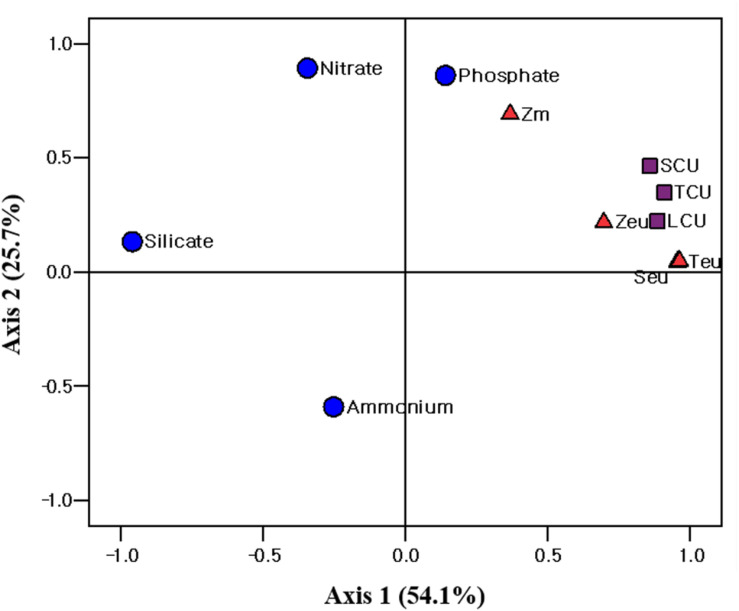
Principle component analysis (PCA) ordination plots of axes 1 and 2 showing carbon uptake rate of different size phytoplankton in relation to environmental variables in the western EJS during the 2016 cruise. T_eu_, water temperature averaged over the depth of the euphotic zone (Z_eu_); S_eu_, salinity averaged over Z_eu_; Z_m_, mixed layer depth; TCU, total carbon uptake rate; SCU, small carbon uptake rate; LCU, large carbon uptake rate.

Principal component analysis (PCA; SPSS 12.0) was conducted to evaluate the relationship between the environmental conditions and carbon uptake rates of the phytoplankton. The first two ordination axes of the PCA explained 79.8% of the carbon uptake rates of the total, small and large phytoplankton communities relative to the environmental conditions during our research period (Figure 7). Water temperature, salinity, and euphotic depth were positively correlated with the carbon uptake rates of the total, small, and large phytoplankton communities (Figure 7). The effect of nutrients on the carbon uptake rates of the entire phytoplankton group was not significant during the study period (Figure 7).

### Biochemical Compositions of the Phytoplankton in the UB and NES

**FIGURE 8:**
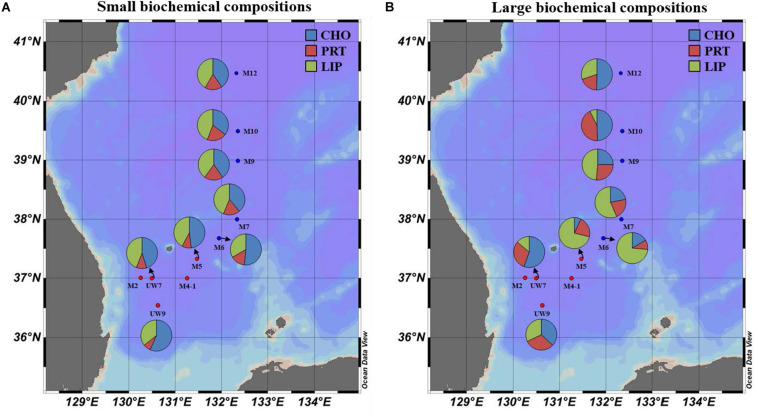
Spatial distributions of biochemical compositions (carbohydrates, proteins, and lipids) for **(A)** small and **(B)** large size phytoplankton in the western EJS in 2016. CHO, carbohydrates; PRT, proteins; LIP, lipids.

The concentrations of the biochemical components (CHO, PRT, and LIP) in both regions are summarized in Table 2. The relative abundances of the biochemical components in the small and large phytoplankton communities in the UB and NES were averaged from the surface to 1% light depth to detect their spatial variation (Figure 8). In the UB, the average compositions of CHO, PRT, and LIP in the small phytoplankton were 52.7 ± 7.9% (range: 41.8–63.0%), 9.2 ± 4.8% (range: 2.3–17.1%), and 38.1 ± 4.3% (range: 33.0–44.2%), respectively, whereas those of the large phytoplankton were 33.4 ± 16.9% (range: 8.8–54.5%), 30.8 ± 10.5% (range: 19.4–49.4%), and 35.8 ± 19.5% (range: 17.8–71.8%) (Figure 8 and Table 2), respectively. In the NES, the CHO, PRT, and LIP contents in the small phytoplankton comprised 42.8 ± 7.6% (range: 28.3–55.5%), 17.5 ± 4.0% (range: 10.7–22.8%), and 39.7 ± 6.5% (range: 31.1–49.4%), respectively, while the average proportions of three biochemical components (CHO, PRT, and LIP) within the large phytoplankton were 28.2 ± 16.3% (range: 6.1–53.3%), 19.6 ± 7.3% (range: 4.3–28.0%), and 52.2 ± 18.9% (range: 18.7–74.3%), respectively (Figure 8 and Table 2).

**TABLE 2:** Concentrations of biochemical compositions, food materials, and calorific contents in the western EJS during the 2016 cruise.

			**Small size phytoplankton**					**Large size phytoplankton**				
				
**Region**	**Station**	**light depth**	**CHO (mg m^–3^)**	**PRT (mg m^–3^)**	**LIP (mg m^–3^)**	**FM (mg m^–3^)**	**Cal (Kcal m^–3^)**	**CHO (mg m^–3^)**	**PRT (mg m^–3^)**	**LIP (mg m^–3^)**	**FM (mg m^–3^)**	**Cal (Kcal m^–3^)**
Ulleung Basin (UB)	M5	100	56.1	10.2	47.5	113.8	0.74	32.8	30.5	54.8	118.1	0.82
	M5	30			N/A					N/A		
	M5	1	55.7	12.1	53.9	121.7	0.81	16.4	36.4	134.6	187.5	1.55
	UW7	100	176.8	14.5	94.1	285.4	1.70	37.8	57.1	20.6	115.6	0.67
	UW7	30	50.9	20.8	50.2	121.9	0.80	149.3	71.2	53.3	273.9	1.51
	UW7	1			N/A					N/A		
	UW9	100	115.9	4.3	63.7	183.9	1.10	29.2	69.7	73.9	172.7	1.20
	UW9	30	78.0	17.9	51.2	147.2	0.91	103.3	56.1	71.6	230.9	1.41
	UW9	1	86.0	13.9	59.8	159.7	1.00	71.4	45.0	31.4	147.9	0.84
North western EJS (NES)	M6	100	81.3	19.6	45.6	146.5	0.87	19.4	32.1	148.8	200.4	1.67
	M6	30	85.1	18.1	54.1	157.4	0.96	42.0	27.3	191.2	260.5	2.14
	M6	1	71.6	32.2	54.6	158.4	0.99	53.4	10.1	172.8	236.3	1.92
	M7	100	71.1	27.9	49.3	148.3	0.91	35.7	64.8	234.3	334.8	2.73
	M7	30	71.9	19.0	86.3	177.1	1.22	76.3	65.9	126.9	269.0	1.88
	M7	1	38.8	30.7	67.7	137.2	0.97	86.8	63.6	151.4	301.8	2.14
	M9	100	74.4	41.8	101.4	217.5	1.50	116.9	101.4	158.2	376.5	2.54
	M9	30	79.2	32.0	75.5	186.7	1.22	100.3	79.6	138.5	318.3	2.16
	M9	1	74.6	38.5	55.6	168.8	1.05	14.0	62.1	152.3	228.4	1.85
	M12	100	68.5	27.1	64.2	159.8	1.04	120.3	45.2	92.0	257.5	1.62
	M12	30	63.8	33.4	63.9	161.1	1.05	78.7	41.3	27.7	147.7	0.81
	M12	1	52.9	22.0	61.9	136.8	0.93	95.8	27.5	57.0	180.3	1.09

*CHO, carbohydrates; PRT, proteins; LIP, lipids; FM, food material; Cal, calorific content; N/A, not available.*

The calorific contents averaged from the euphotic depths in the UB ranged from 0.74 to 1.70 Kcal m^–3^, with a mean of 1.01 ± 0.33 Kcal m^–3^, for the small phytoplankton and from 0.67 to 1.55 Kcal m^–3^, with a mean of 1.14 ± 0.36 Kcal m^–3^, for the large phytoplankton (Table 2). The average calorific contents in the NES ranged from 0.87 to 1.50 Kcal m^–3^ (1.06 ± 0.18 Kcal m^–3^) for the small phytoplankton and from 0.81 to 2.73 Kcal m^–3^ (1.88 ± 0.54 Kcal m^–3^) for the large phytoplankton (Table 2).

## Discussion

### Spatial Distributions of Size-Fractionated Chl *a* Concentration in the Two Different Regions of the Western EJS

The average total chl *a* concentrations in both regions were similar; the phytoplankton community compositions differed slightly by size between the UB and the NES (Figure 5A and Table 1), although no significant difference was found. Based on the size-fractionated chl *a* concentration, small phytoplankton (55.6 ± 9.6%) had a relatively higher contribution to the total biomass in the UB, whereas the contribution of large phytoplankton (51.4 ± 11.3%) was slightly higher than that of small phytoplankton in the NES (Table 1).

Noticeable patterns in the spatial distributions of the chl *a* concentrations of small and large phytoplankton integrated over the depths sampled were not found in the UB, whereas the integrated chl *a* concentration of the large phytoplankton in the NES increased with latitude, with the lowest value at M6 (7.7 mg chl *a* m^–2^) and the highest value at M12 (27.7 mg chl *a* m^–2^) (Figure 5A and Table 1). The spatial variations in the concentration of chl *a* from the large phytoplankton integrated from the surface to a light depth of 1% in the NES were mostly related to temperature (Pearson’s *r* = –0.93, *p* < 0.05) and nitrate concentration (Pearson’s *r* = 0.90, *p* < 0.05) during the study period. Phytoplankton community size structure is sensitive to environmental conditions. According to previous studies, the size compositions of phytoplankton assemblages in the ocean can be affected by water temperature (Morán et al., 2010; Mousing et al., 2014) and nutrient availability (Agawin et al., 2000; Finkel et al., 2005, 2007; Marañón et al., 2012). Increasing water temperature increases the metabolic rate of phytoplankton (Gillooly et al., 2001), which increases resource requirements and, therefore, competition for nutrients (Mousing et al., 2014). In addition, warming temperatures increase the development of water stratification, resulting in nutrient depletion in the euphotic layer (Calvo-Díaz and Morán, 2006; Marañón et al., 2012). In other words, both the increasing cellular nutrient demands due to high metabolic rates as a function of temperature and a reduction in upward nutrient concentration in the euphotic layer due to water stratification can be expected to cause increasing resource competition and a smaller community mean cell size (Mousing et al., 2014). Among the major inorganic nutrients in the ocean, source of nitrogen (e.g., nitrate and ammonium) are the main factors controlling phytoplankton cell size (Stolte et al., 1994; Stolte and Riegman, 1995). In general, large phytoplankton tend to prefer nitrate, which is abundant in upwelling areas, in coastal areas in early spring, and on continental shelves, whereas ammonium is favored by small phytoplankton (Dauchez et al., 1996; Stolte and Riegman, 1995). Indeed, relatively low temperatures and high nitrate concentrations were generally observed in the higher latitudes of the NES during our research period (Table 1).

Overall, the average total chl *a* concentrations in the UB (31.0 mg chl *a* m^–2^) and NES (28.4 mg chl *a* m^–2^) during the spring season of 2016 were lower than those reported previously in the EJS (Kang et al., 2004; Lee et al., 2017b). During the spring bloom in the UB during April 2001, a high chl *a* concentration (43.8 mg chl *a* m^–2^) was observed (Kang et al., 2004). During this period, autotrophic nanoflagellates and picoeukaryotes were the main components of the total chl *a* (Kang et al., 2004). This finding was similar to our results, which showed that the contribution of small phytoplankton to the total chl *a* was high (55.6%) in the UB. In the northern EJS, including the NES region in spring 2015, Lee et al. (2017b) reported significantly higher chl *a* concentrations (84.6 mg chl *a* m^–2^), which were predominantly (47.7–72.5%) due to large phytoplankton. In contrast, the chl *a* concentration (28.8 mg chl *a* m^–2^) in the shelf region of the southern East China Sea (Chen, 2000) is similar to those in the UB and NES during our research period, and the size compositions (small: 57.4% and large: 42.6%) in the shelf region are close to those in the UB in this study. Currently experiencing rapid environmental changes, the northern Chukchi Sea in the Arctic Ocean also had similar chl *a* concentrations (30.5 mg chl *a* m^–2^) and size compositions (small: 55.1% and large: 44.9%) (Yun et al., 2015) as those found in this study. The relatively high contributions of small phytoplankton to the total community in the shelf regions of the southern East China Sea and the northern Chukchi Sea are known to result from depleted nitrogen sources, especially nitrate (Chen, 2000; Yun et al., 2015).

### Difference in the Carbon Uptake Rate Between the UB and NES

Based on the chl *a* concentration, the total phytoplankton biomass was not significantly different between the UB (31.0 ± 8.4 mg chl *a* m^–2^) and NES (28.4 ± 7.9 mg chl *a* m^–2^) during our research period. However, the hourly carbon uptake rate of the total phytoplankton community in the UB (79.0 ± 12.2 mg C m^–2^ h^–1^) was approximately two times higher (*t*-test, *p* < 0.01) than that in the NES (40.7 ± 2.2 mg C m^–2^ h^–1^) (Figure 6 and Table 1). Primary production can be affected by various physicochemical and biological factors, such as temperature, light availability, ambient nutrient concentrations, phytoplankton community structure, and grazing pressure (Kwak et al., 2014; Lee et al., 2020). Based on the PCA, temperature and euphotic depth were major controlling factors for the total and size-fractionated carbon uptake rates in both regions during the observation period (Figure 7). Lewandowska et al. (2012) found strong relationships between primary production by phytoplankton and temperature. Primary production had positive correlations with water temperature when nutrient concentrations and light availability were not limiting, whereas increased water temperature under unsaturated light conditions, which limits the carbon incorporation process, led to decreased primary production due to the enhancement of grazing activity and community respiration (Lewandowska et al., 2012). Other studies have also reported similar results on the positive effects of temperature on the photosynthesis (Andersson et al., 1994) and growth rate (Rhee and Gotham, 1981) of phytoplankton. In general, phytoplankton spring blooms start when light intensity increases in the upper water column through the development of stratification after well-mixed conditions during the winter (Huisman et al., 1999; Jo et al., 2007). This means that the main controlling factor for phytoplankton blooms in the spring season is not nutrients but light, since the major nutrients required for photosynthesis are made available by the mixing of the water column by wind in the winter season. Light availability might also not be a main limiting factor for photosynthesis during the peak timing of spring blooms. Indeed, the maximum carbon uptake rates during the study period were observed at the surface layer (100%–50% light depth: 0–8 m; Table 1). Therefore, the colder water temperature in the NES (7.9 ± 2.5°C) compared to that in the UB (13.4 ± 0.8°C) could have a negative effect on the phytoplankton community in terms of photosynthesis (i.e., carbon uptake rate). During the study period, the deeper euphotic depth in the UB than in the NES could have enabled the light to penetrate deeper, allowing more phytoplankton within the euphotic water column to photosynthesize. This can be another reason for the higher integrated carbon uptake rate of phytoplankton in the UB than in the NES.

Based on the average daily carbon uptake rate in this study, the estimated annual primary production in the UB and NES was 284 g C m^–2^ y^–1^ and 147 g C m^–2^ y^–1^, respectively. The annual production in both regions during our research period is consistent with previous studies in the UB (273 g C m^–2^ y^–1^ and 280 g C m^–2^ y^–1^) (Kwak et al., 2013; Joo et al., 2014) and in the northern EJS (159 g C m^–2^ y^–1^), including the NES regions (Lee et al., 2017b). The annual primary production in the UB (a deep basin with a water depth > 2,000 m) was markedly higher than that in oceanic regions and other basins deeper than 200 m, whereas the annual primary production in the NES was similar to that in these regions. The annual primary production in oceanic regions was generally low, with a range from 55 to 102 g C m^–2^ y^–1^, whereas upwelling regions had considerably higher primary production rates (300–398 g C m^–2^ y^–1^) (Joo et al., 2014; references therein). In the eastern and western basins of the Mediterranean Sea, which have environmental conditions similar to those in the EJS, the annual carbon uptake rates were 109 g C m^–2^ y^–1^ and 158 g C m^–2^ y^–1^, respectively (Estrada, 1996). Because of its high productivity compared to those in other oceanic regions, the UB is considered a prominent biologically productive region and is referred to as a “hotspot” in the EJS (Kwak et al., 2013; Joo et al., 2014). This hot-spot is sustained by several potential mechanisms, such as different types of subpolar fronts (Chiba et al., 2008), frequent eddies (Hyun et al., 2009; Kim et al., 2012; Lim et al., 2012), and coastal upwellings (Yoo and Park, 2009).

### Contributions of Small and Large Phytoplankton to the Total Carbon Uptake Rate

The contributions of small and large phytoplankton to the total carbon uptake rate were different from their contributions to the chl *a* in both regions. The large phytoplankton (mean ± *SD* = 54.8 ± 3.7%) had a higher contribution than the small phytoplankton (45.2 ± 3.7%) to the total carbon uptake rate of the total phytoplankton in the UB. In the NES, the contribution of large phytoplankton (60.5 ± 7.8%) to the total carbon uptake rate was significantly higher (*t*-test, *p* < 0.01) than that of small phytoplankton (39.5 ± 7.8%) (Figure 5 and Table 1). The different contributions of small and large phytoplankton to the total chl *a* and carbon uptake rate during spring 2016 could have been caused by several environmental conditions (i.e., temperature and euphotic depth), as mentioned above. In the UB, the contribution of small phytoplankton to the total primary production for our cruise period (45.2%; spring season) was higher than that in July (35%; summer season) (Kwak et al., 2014). In general, small phytoplankton are predominant under warming conditions (Morán et al., 2010; Mousing et al., 2014). The low contribution of small phytoplankton observed in the UB, even in the summer season (Kwak et al., 2014), could be the result of dynamic environmental conditions (i.e., upwelling, eddies, and fronts), which allow the UB to have higher primary productivity than other oceanic regions and relatively constant primary production among different months and years (Lee et al., 2017b). Indeed, Kwak et al. (2014) measured a relatively high primary production rate (716 mg C m^–2^ d^–1^) even in the summer season (June–August), when phytoplankton are not normally actively growing at other temperate locations (Lee et al., 2017b). The value observed in summer by Kwak et al. (2014) is comparable to the primary production rate observed during the spring bloom in this study (790 mg C m^–2^ d^–1^).

**FIGURE 9:**
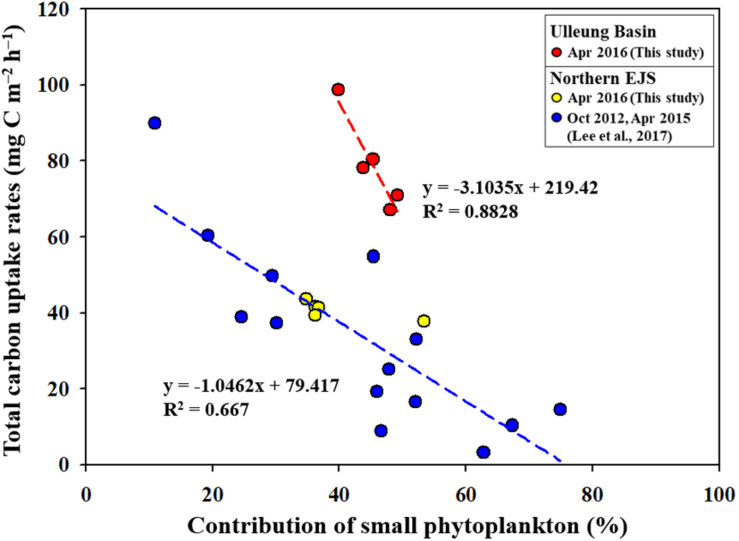
Relationships between productivity contributions of small phytoplankton and the total primary productions in the UB and northern EJS.

According to previous studies (Agawin et al., 2000; Morán et al., 2010; Hilligsøe et al., 2011; Mousing et al., 2014), recent climate changes, especially warming temperatures, are expected to increase the contribution of small phytoplankton to the total phytoplankton community, which enhances the importance of small phytoplankton as a basic food source in marine ecosystems. Indeed, negative correlations between total primary production and small phytoplankton contributions were consistently observed in the Chukchi Sea (unpublished data) and the Amundsen Sea (Lee et al., 2017c), which have experienced rapid climate change. Lee et al. (2017b) reported a negative relationship between the small phytoplankton contribution and the total primary production in the northern EJS, which has been experiencing a drastic increase in sea surface temperature for several decades (Kim et al., 2001). We also found a marked decreasing trend in the total carbon uptake rates with increasing contributions of small phytoplankton in the UB (Figure 9). In addition, our data points observed in the NES are consistent with the regression line reported in Lee et al. (2017b). An interesting feature is that the rate of decrease in the total carbon uptake rate with increasing contributions of small phytoplankton is considerably faster (by approximately three times) in the UB than in the northern EJS (Figure 9). This is very meaningful, as it could indicate that the primary production in the UB, a biological hotspot in the EJS, might respond more sensitively to ongoing warming conditions.

### Physiological Status and Food Quality of Phytoplankton in the Two Different Regions

The biochemical compositions of phytoplankton provide important information about their physiological status (Kang et al., 2017; Lee et al., 2020). According to previous studies, biochemical compositions are affected by environmental factors, such as nutrient concentrations (Morris et al., 1974; Kilham et al., 1997) and growth stages (Smith et al., 1987; Kang et al., 2017). The relative proportions of LIP and CHO, known as storage compounds, tend to be high under nutrient deficient conditions (especially when nitrogen sources are limiting) (Yun et al., 2015; Kim et al., 2016; Kang et al., 2017) and during stationary growth phases (Morris, 1981; Ríos et al., 1998; Kang et al., 2017), whereas the allocation of PRT increases when there are abundant nutrient resources (DiTullio and Laws, 1983; Palmisano et al., 1988). Overall, the biochemical composition of the small phytoplankton during the study period as dominated by CHO (UB: 52.7 ± 7.9%; CE: 42.8 ± 7.6%), followed by LIP (UB: 38.1 ± 4.3%; CE: 39.7 ± 6.5%) in both regions (Figure 8A and Table 2). The small phytoplankton in the UB may have experienced a limitation in nutrient uptake due to competition with large phytoplankton during the study period. Large phytoplankton, such as diatoms, have a competitive advantage over small cells in nutrient-sufficient conditions due to their higher nutrient uptake rate (Litchman et al., 2007; Marañón et al., 2012) and accumulation ability (Thingstad et al., 2005; Marañón et al., 2012). In a parallel study on the species compositions of phytoplankton (unpublished data), the major classes for large phytoplankton in the UB were diatoms (28.5%) and cryptophytes (25.6%) which are known to assimilate significantly higher biochemical components during the active growth period with sufficient nutrients (Moal et al., 1987); small phytoplankton in the UB consisted of the prasinophytes (19.5%), prymnesiophytes (13.6%), and cyanophytes (7.2%) during our study period. Roy (2018) also reported that the contribution of large phytoplankton to the biochemical components is higher in coastal regions, which generally have sufficient nutrient conditions due to river input and coastal upwelling. In the NES, the small phytoplankton which mostly consisted of prasinophytes (11.7%), prymnesiophytes (11.5%), and cyanophytes (13.3%) (unpublished data) may have had low metabolic rates (Gillooly et al., 2001) and been in a stationary growth phase (Rhee and Gotham, 1981) due to the cold water temperature, resulting in the high CHO and LIP contents observed during the study period. The physiological status of the large phytoplankton in the UB, except M5, appears to be better than that in NES (diatoms: 34.4% and cryptophytes: 16.5%), since the contributions of PRT contents to the total biochemical compositions were relatively high in UW7 (37.7 ± 10.0%) and UW9 (31.7 ± 12.3%) (Figure 8B and Table 2). A potential reason for the relatively large contribution of PRT by the large phytoplankton in the UB is that they were in an active growth phase with an increased nutrient uptake rate because of the warmer water temperature, contrary to large phytoplankton in the NES (Rhee and Gotham, 1981; Lee et al., 2009). On the other hand, the large phytoplankton in M5 had significantly higher LIP contents than those at other sites (Figure 8B and Table 2), although the highest carbon uptake rate was observed during our cruise period (Figure 5B and Table 1). This unexpected observation might have resulted from a deficiency in nitrogen and phosphate sources, as they had already been exhausted by phytoplankton photosynthesis. de Madariaga and Joint (1992) reported that phytoplankton under nitrogen- (nitrate and ammonium) and phosphate-limited conditions had high LIP concentrations. At station M5, nitrogen, especially nitrate, and phosphate concentrations were lower than those at the other stations in the UB (Table 1). However, we are uncertain whether consumption or other mechanisms drove this nutrient deficiency; therefore, more research and data are needed to understand the exact mechanism for the mismatch between the productivity and physiological status of the phytoplankton in M5 during our research period. In the NES, the large phytoplankton had relatively high lipid compositions, except at site M12, where the dominant component was CHO (Figure 8B and Table 2). The physiological status of these large phytoplankton seemed to suggest that they were in a stationary growth phase caused by cold water temperatures, as mentioned above (Rhee and Gotham, 1981; Lee et al., 2009; Kwak et al., 2014).

The average calorific contents in the UB were similar between the small (1.01 ± 0.33 Kcal m^–3^) and large (1.14 ± 0.36 Kcal m^–3^) phytoplankton, whereas those in the NES were higher (*t*-test, *p* < 0.01) for the large phytoplankton (1.88 ± 0.54 Kcal m^–3^) than the small phytoplankton (1.06 ± 0.18 Kcal m^–3^) (Table 2). There was no spatial difference in the calorific value for the small phytoplankton between the UB and the NES (Table 2). In contrast, the calorific values of the large phytoplankton in the UB were significantly lower (*t*-test, *p* < 0.01) than those in the NES, although the total primary production was approximately two times lower in the NES than in the UB (Table 2). This pattern was caused mainly by the difference in the amount of FM, especially LIP, in the large phytoplankton between the UB and the NES (Table 2). There was no significant difference in the average CHO (UB: 62.9 ± 48.3 mg m^–3^; NES: 70.0 ± 36.5 mg m^–3^) or PRT (UB: 52.3 ± 15.7 mg m^–3^; NES: 51.7 ± 25.8 mg m^–3^) concentrations in the large phytoplankton between the two regions (Table 2). In contrast, the average LIP concentration of the large phytoplankton was significantly higher (*t*-test, *p* < 0.01) in the NES (137.6 ± 56.6 mg m^–3^) than in the UB (62.9 ± 37.1 mg m^–3^) (Table 2). Moreover, the higher energy content of LIP compared to other components led to the significant difference in the calorific value between the UB and the NES. Therefore, the relatively low primary production rate in the NES can be compensated by high calorie LIP-dominant FM.

Considering the importance of small phytoplankton, which will contribute increasing amounts to the total biomass under warming conditions (Morán et al., 2010), we assessed the calorific value per small and large phytoplankton cell by dividing the calorific content by the chl *a* concentration (Table 3). Overall, the calorific content per unit of chl *a* (hereafter Cal/chl) was higher for the large phytoplankton than the small phytoplankton in both regions (Table 3). Furthermore, the contribution of the large phytoplankton to the Cal/chl of the total phytoplankton was higher than that of the small phytoplankton in both regions (Table 3). This means that large phytoplankton could be more efficient as a food source, providing a higher energy value per unit to organisms in higher trophic levels. This finding is in contrast to the previous results from Kang et al. (2017) and Kim et al. (2019), who observed that small phytoplankton assimilated more FMs and energy per unit of chl *a* concentration. These inconsistent results might be caused by different research regions and periods. Kang et al. (2017) conducted their research in the northern EJS during the post-spring bloom period in 2015, when the phytoplankton had entered a stationary growth phase, whereas our study was conducted during the peak of the spring bloom based on satellite ocean color data provided in a parallel study (unpublished data). Kim et al. (2019) carried out their study in Gwangyang Bay, South Korea, which is largely affected by river inputs.

**TABLE 3:** Comparison of Cal/chl ratios between small and large phytoplankton.

**Region**	**Season**	**Contents**	**Phytoplankton size**			**Ratio**		**References**
				
			**Small**	**Large**	**Total**	**Small: Total**	**Large: Total**	
Northern East/Japan Sea	Spring and fall	Cal. (Kcal m^–3^)	0.60	0.80	1.40	0.43	0.57	Kang et al., 2017
		Chl *a* (mg m^–3^)	0.60	1.70	2.30	0.26	0.74	
		Cal/Chl *a* (Kcal mg^–1^)	1.00	0.47	0.61	1.64	0.77	
Southern coastal areas in Korea: Gwangyang Bay	Four seasons	Cal. (Kcal m–3)	1.70	2.10	3.80	0.45	0.55	Kim et al., 2019
		Chl *a* (mg m–3)	0.80	2.50	3.30	0.24	0.76	
		Cal/Chl *a* (Kcal mg^–1^)	2.13	0.84	1.15	1.85	0.73	
South western East/Japan Sea: Ulleung Basin (UB)	Spring	Cal. (Kcal m^–3^)	1.01	1.14	2.15	0.47	0.53	This study
		Chl *a* (mg m^–3^)	0.52	0.45	0.97	0.54	0.46	
		Cal/Chl *a* (Kcal mg^–1^)	1.92	2.56	2.21	0.87	1.15	
Northwestern East/Japan Sea (NES)	Spring	Cal. (Kcal m^–3^)	1.06	1.88	2.94	0.36	0.64	
		Chl *a* (mg m^–3^)	0.59	0.69	1.27	0.46	0.54	
		Cal/Chl *a* (Kcal mg^–1^)	1.81	2.74	2.31	0.78	1.19	

*Cal/chl, calorific content per unit of chl *a*.*

## Summary and Conclusion

Under current climate changes, an increase in the contribution of small phytoplankton to the total phytoplankton community has been observed in various oceans (Agawin et al., 2000; Li et al., 2009; Morán et al., 2010; Hilligsøe et al., 2011; Mousing et al., 2014; Joo et al., 2017), which indicates a growing importance of small size phytoplankton as a basic food source for higher trophic level organisms in the marine ecosystems. This study in the two different regions (i.e., UB and NES) of the western EJS reported the influence of small phytoplankton contribution to the primary production and different energy efficiencies between small and large phytoplankton based on their biochemical components. According to previous studies (Lee et al., 2017b,c), the total primary production could be decreased by the increase of small phytoplankton contribution. Indeed, this study also proved a negative correlation between the total carbon uptake rates and the contribution of small phytoplankton in the UB and NES (Figure 9). In particular, the decreasing trend of the total carbon uptake rates in the UB under increasing small phytoplankton contributions is more faster in this study (Figure 9) compared to previous studies in the polar regions (the Chukchi Sea–unpublished data; the Amundsen Sea–Lee et al., 2017c.) and the northern EJS (Lee et al., 2017b) which have been experiencing drastic environmental changes. It means that the primary production in the UB as a biological hot spot of the EJS could be responded sensitively to ongoing climate changes, especially warming water temperature. However, there are some uncertainties for that since only a few data points were available in the UB in this study. Further evaluation for the rapid decreasing trend in the total primary production with increasing small phytoplankton contribution should be conducted in the UB. Therefore, long term observations for the seasonal and annual primary productions and contributions of small phytoplankton in the EJS, including the UB, are needed for a better understanding of potential ecosystem change under ongoing climate change.

In terms of energy efficiency in differential size phytoplankton, large phytoplankton could provide higher energy value per unit cell than small phytoplankton based on the Cal/chl of the two different cell-sized of phytoplankton in the UB and NES during this study (Table 3). In contrast, previous studies found opposite results that small phytoplankton had relatively higher Cal/chl than large phytoplankton (Kang et al., 2017; Kim et al., 2019). This inconsistency between this and other studies might be resulted from different research regions and periods. Further studies for the contrasting patterns are necessary to understand the subsequent nutritional effects of small phytoplankton as a potential food source on higher trophic levels in a projected warmer oceanic condition.

## Data Availability Statement

The original contributions presented in the study are included in the article/supplementary material, further inquiries can be directed to the corresponding author/s.

## Author Contributions

SL conceived the ideas and designed the methodology. JK performed the field experiments, data analysis, and wrote the manuscript. HJ and JLe performed the field experiments and conducted the lab experiment. J-HLi and CL conducted the lab experiment. DL and HB analyzed the satellite data. C-KK critically reviewed the manuscript. All authors contributed to the article and approved the submitted version.

## Conflict of Interest

The authors declare that the research was conducted in the absence of any commercial or financial relationships that could be construed as a potential conflict of interest.
